# Leveraging design thinking to tackle contemporary admissions challenges in health professions education

**DOI:** 10.1371/journal.pone.0352192

**Published:** 2026-06-23

**Authors:** Adina O. Davidson, Wendy C. Cox, Heather Azzu, Kelly Womack-Adams, Jacqueline E. McLaughlin

**Affiliations:** 1 Center for Innovative Pharmacy Education and Research, UNC Eshelman School of Pharmacy, The University of North Carolina, Chapel Hill, North Carolina, United States of America; 2 Division of Practice Advancement and Clinical Education, UNC Eshelman School of Pharmacy, The University of North Carolina, Chapel Hill, North Carolina, United States of America; 3 Office of Recruitment and Admissions, UNC Eshelman School of Pharmacy, The University of North Carolina, Chapel Hill, North Carolina, United States of America; 4 Department of Teaching, Learning and Curriculum, Reich College of Education, Appalachian State University, Boone, North Carolina, United States of America; University of Foggia: Universita degli Studi di Foggia, ITALY

## Abstract

**Purpose:**

This study illustrates the use of design thinking (DT) as a structured, participatory approach to explore contemporary complex challenges in health professions admissions, including the rise of generative AI, remote interviews, and the elimination of standardized admissions tests. This work focuses on stakeholder-driven problem framing and idea generation rather than evaluating outcomes.

**Methods:**

A two-hour workshop engaged 15 purposively sampled stakeholders, including faculty, staff, application readers, and student ambassadors, in collaborative problem framing, ideation, and rapid prototyping of admissions concepts. Generative artifacts (brainstorming outputs, reflection worksheets, facilitator notes) and post-session surveys were analyzed using thematic synthesis and descriptive statistics to characterize emergent ideas and participant perspectives.

**Major findings:**

Participants generated ideas that clustered into three major themes: interview restructuring, GenAI integration and compliance, and broadening of admissions criteria, illustrating how stakeholders reframed challenges and proposed diverse solution pathways. Survey responses reflected descriptive indicators of participant experience, suggesting the workshop supported creative problem solving (Mean 4.7 ± 0.5), idea generation (4.8 ± 0.4), and collaboration (4.4 ± 0.5).

**Conclusions:**

Findings suggest that DT offers a structured, iterative framework for stakeholder-driven idea generation and problem reframing in an evolving admissions context. The workshop demonstrates the potential of collaborative, reflective processes to surface assumptions and generate diverse perspectives to inform future exploration of admissions practices.

## Introduction

Design thinking (DT), also referred to as human-centered design, user-centered design, and design innovation, is a problem-solving framework suited to complex, ambiguous challenges. Grounded in creativity and cognition, DT emphasizes iterative cycles of inspiration, empathizing with stakeholders and defining problems; ideation, generating and refining ideas; and implementing, introducing and improving the derived solution [1]. As a structured yet flexible approach, DT supports collaboration, surfaces assumptions, and enables stakeholders to reframe entrenched problems [1]. Within health professions education, DT has been utilized to explore various curricular and organizational questions, such as redesigning clinical rotations, identifying barriers to rural experiential education, and exploring pathways to healthcare administration [2–4].

Student selection in health professions education represents one such complex institutional challenge. Assessing the suitability of candidates for our programs is one of the most important tasks undertaken by health professions schools. The issue of student selection – that is, predicting who is most likely to succeed in a curriculum and become a successful practitioner – serves as the foundation of our healthcare system. As such, schools must develop robust selection processes that comply with professional standards, align with institutional values, and adapt to the ongoing changes facing healthcare and higher education [5].

Over the past decade, selecting students for health professions programs has become increasingly complex. Student selection has been influenced by various social, legal, economic, and technological changes, including the US Supreme Court ruling on affirmative action, shifting enrollments and projected workforce shortages, procedural adjustments in response to the COVID-19 pandemic, controversy surrounding the use of standardized admissions tests, and the advent of generative artificial intelligence (GenAI) [6–9]. In response to these challenges, scholars have advocated for expanding, updating, and enhancing our selection strategies [6,7]. In this context, admissions processes must balance institutional values, professional standards, and uncertainty, often with limited guidance on how to adapt [5,10]. As noted by Conrad and colleagues, “Selection — who gains access to a medical education and to a career as a physician, researcher, and/or faculty member — is as much art as science*.*” (11 p. 1472) Innovating our admissions processes to address these contemporary challenges will require creative thinking rooted in localized context [10,11].

This paper presents an exploratory, practice-based case study illustrating how DT can be applied to student selection in a health professions program. Rather than evaluating the effectiveness of an intervention, the aim is to document DT as a structured, participatory approach to stakeholder engagement, problem reframing, and idea generation within a localized admissions context. Specifically, we describe the design and implementation of a DT workshop at the University of North Carolina at Chapel Hill (UNC) Eshelman School of Pharmacy and examine the types of ideas and perspectives that emerged through this process. Framed as generative, workshop-based inquiry, this study draws on artifacts of co-creation (e.g., brainstorming outputs, notes, and reflections) to provide a process-oriented insight into how DT can be used to explore complex institutional challenges and inform future phases of prototyping, testing, and evaluation.

## Methods

### Context of workshop

The current admissions process for the Doctor of Pharmacy (PharmD) degree program at the UNC Eshelman School of Pharmacy consists of three stages: stage 1 = academic review to ensure candidates have met the required pre-requisite courses and minimum grade point average (GPA) for application to the program; stage 2 = a comprehensive application review completed by two independent reviewers using a rubric focused on co-curricular experiences, leadership experience, community service, research, and overall life experiences [5]; and stage 3 = interviews using the multiple mini-interview (MMI) to evaluate constructs considered important for success in the program [6].

The School’s admissions process underwent several modifications in response to the COVID-19 pandemic starting in 2020. These modifications included quickly moving candidate interviews to a remote platform, which have continued post-pandemic to reduce barriers and increase efficiency, such as limiting time off from school or work, lowering expenses related to travel, and meeting candidate preference for virtual interviews [7]. In 2020, the School decided to make the standardized test for pharmacy school admissions, Pharmacy College Admissions Test (PCAT), optional for candidates. While this decision was made to provide flexibility during the pandemic due to testing site closures and travel issues, it subsequently limited the data available to evaluate student writing and compare candidates across undergraduate institutions utilizing standardized data. In 2024, the PCAT was discontinued by Pearson Assessments US, making the test unavailable for any candidate or school.

New challenges, such as changes and opportunities associated with GenAI, have further prompted the School’s admissions team to consider additional modifications. As such, a team consisting of the Associate Dean for Admissions and Accreditation, Director of Admissions, Director of the School’s Center for Innovative Pharmacy Education Research (CIPhER), and two postdoctoral research fellows focused on educational innovation, was formed to explore options for addressing these challenges.

### Design of workshop

Members of the team met to discuss the need for potential modifications to the School’s admissions process. Through this discussion, it was determined that dedicated time with various participants in the admissions process, such as application reviewers and admissions committee members, was needed to generate creative ideas that may improve the admissions process. One team member with DT expertise offered to design and lead a 2-hour workshop that focused on generating and critically evaluating potential solutions.

For the purpose of this workshop, the “user” was determined to be individuals who played a defined role in the admissions process. As such, workshop participants were strategically selected based on their role in the PharmD admissions process at the School, including faculty who served on the School’s PharmD Recruitment and Admissions Committee (n = 8), recruitment and admissions team staff (n = 4), application reviewers (n = 2), and student Recruitment and Admissions Program (RAP) ambassadors in the third year of the PharmD program (n = 3). This group represented 80% of the Committee faculty, 100% of the recruitment and admissions team, 100% of the application reviewers, and 60% of the invited RAP ambassadors. Faculty ranged in service to the Committee from <1 year to >15 years. One faculty member was tenured and the others were fixed-term. Four of the faculty participants were also alumni of the PharmD program. Two authors served as facilitators during the brainstorming session and one author served as a participant.

The team designed the workshop to include three DT steps: defining admissions challenges (*inspiration*), brainstorming solutions (*ideation*), and prototyping strategies through critical reflection (*implementation*). A 5-minute word association warm-up was used to help prepare participants for creative thinking. After the warm-up, participants were divided into three groups, each consisting of a mix of individuals with various admissions roles.

#### Inspiration.

Each group was randomly provided one of three pre-identified admissions topics that could be used to inspire brainstorming (i.e., launching points). Launching points were pre-identified by the workshop team based on recent discussions among the Committee and admission team staff. These points were inspired primarily by recent experiences and pain points during recent admissions cycles, such as the loss of the PCAT as a source of reliable comparisons between candidates. The workshop team prioritized launching points they believed to be strategic and feasible to address during the workshop. Each group was provided time to review and revise the statement to ensure its accuracy and relevance to the users:

- Launching point 1: Fair Use of Technology by Candidates. Candidates are utilizing various technologies during the application process (e.g., application, interview). How might we ensure that candidates are using technology in fair and ethical ways?
- Launching point 2: Reliable Comparison of Candidates. Candidates have varied levels of experience, knowledge, and preparedness for pharmacy school. How might we reliably compare candidates in a post-PCAT world?
- Launching point 3: Strategic use of Technology by Evaluators/Admissions Office. Our admissions process utilizes various technologies for the admissions process (e.g., online application, Kira talent). How might we strategically utilize current and emerging technologies (e.g., GenAI) in the admissions process?

#### Ideation.

Once the launching points were refined, participants were provided five minutes to individually brainstorm and write down strategies for addressing the launching point. Divergent thinking was prioritized, and participants were reminded that within the DT parameters, all contributions were welcomed in this activity. Participants shared their ideas with the group, added additional ideas, identified themes across their ideas, and wrote the themes onto chart paper. A gallery walk was used to allow all workshop participants to review all launching points and ideas, with the option of adding new ideas and demarcating any strategies they found most resonant [8].

#### Implementation.

Following the gallery walk, each participant was provided a reflection worksheet and instructed to select 3–5 ideas from their group chart. Participants were asked to reflect on these ideas in terms of cost, geography, and equity, and identify related pros and cons of each idea. Cost, geography, and equity were selected as design constraints given their relevance to the School’s mission and alignment with the PharmD admissions philosophy. Geography, for example, might represent limited access for some candidates if the School adopted a required in-person interview; alternatively, it might represent expanded access if the School implemented an asynchronous interview process. Cost could be prohibitive for the acquisition of certain new technologies that support the admissions process, or savings for the sunsetting of current technologies. Equity might include the impact of admissions processes on students requiring additional time for real-time assessments. These constraints may also intersect, with GenAI potentially bolstering equity for non-native English speakers and international applicants – enabling them to better understand question prompts or articulate their opinions when writing essays.

Using these constraints, participants completed the worksheet independently, then discussed their evaluations as a group. Based on these discussions, each group drafted 1–3 prototypes for consideration in the admissions process. Prototypes were shared through large group debrief. While this activity did not include actual implementation of a strategy, it provided critical insights into aspects of implementation that may impact the feasibility and effectiveness of a proposed strategy.

### Evaluation of workshop

The in-person workshop was held in person in January 2025 as a structured, participatory ideation exercise. Data consisted of artifacts generated through the DT process, including individual brainstorming outputs, group-generated themes, creative reflection worksheets, and facilitator notes, along with brief post-workshop evaluation survey. The survey consisted of 5 Likert-scale questions ranging from 5-Strongly Agree to 1-Strongly Disagree and an open-text item to assess participants’ experiences. All data were collected anonymously.

Consistent with the study’s exploratory aim, analysis focused on synthesizing emergent ideas rather than evaluating outcomes. Materials were transcribed into an electronic format and analyzed using an inductive selective coding approach and thematic analysis was conducted to identify recurring themes within and across groups. Two coders independently reviewed the data and engaged in iterative discussion to refine themes. Trustworthiness was ensured through triangulation, including transparent documentation of coding procedures, intercoder debriefing, and limited member checking to validate themes. Inter-coder agreement was sought to ensure themes accurately reflected the data. Inter-rater reliability was assessed using percent agreement. The number of items on which the coders assigned the same code was divided by the total number of items coded, and the result was multiplied by 100 to yield the percentage agreement. The inter-rater reliability was 90.5%.

Given the small, purposively selected sample (n = 15) and single-site design, quantitative analysis of post-evaluation survey data from the Likert questions was limited to descriptive statistics. Mean and standard deviation (SD) were calculated and are interpreted as indicators of participant experience rather than evidence of effectiveness. Survey responses are subject to potential response and social desirability biases.

It is important to note that this study did not include implementation or evaluation of the ideas generated. Instead, findings represent illustrative outputs of an early-stage, generative process, highlighting the range of perspectives and potential solutions elicited through a DT approach. A summary of results was compiled into a 2-page summary report that was shared with the School’s admissions committee for member-checking and to support ongoing reflection and future development. This study was determined to be not human subjects research by the UNC at Chapel Hill Institutional Review Board (IRB Number: 24–3149); as such, informed consent was not required or obtained, and all data were collected anonymously.

## Results

Given the exploratory aim, results are presented as illustrative outputs of the DT process rather than evidence of effectiveness. The findings reflect how participants engaged with the workshop to generate, refine, and prioritize ideas within a specific institutional context (e.g., admissions). Of note, the ideated solutions presented in the results are not meant to be an exhaustive list for others to utilize; rather, they demonstrate specific ideas contextualized to the unique experiences, processes, priorities, and resources at our School.

During the ideation phase, participants generated approximately 76 ideas of which 43 were selected across three focal areas for further consideration. Ideas varied widely in scope and specificity. Voting was used as a pragmatic prioritization tool within groups, with selected ideas serving as starting points for further development rather than validated solutions. Examples of the ideas generated included: “have students evaluate an AI response to a scenario” (n = 7 votes), “technology that blocks the internet during interviews” (n = 10 votes) “add writing activity to the end of candidates day” (n = 13 votes), “new interview and application questions” (n = 1 vote), and “use GenAI to develop objective rubrics” (n = 1 vote). Vote counts per ideas ranged from 0–13. Groups prioritized prototypes for further development based on higher vote totals. At the end of the workshop, each of the three groups presented 1–2 prototypes for consideration (4 total prototypes were presented due to limited time), incorporating their suggestions through the perspectives of cost, equity, and geographical factors. As an example, in response to the launching point of *reliable comparison across candidates*, one group recommended incorporating a task-based interview station, such as real-time written essays or math problems that would preclude the use of GenAI. As it related to perspectives of cost, equity and geographical factors, the group opined that the real-time component might allow for fair comparisons across candidates on constructs of interest (e.g., in absence of standardized tests), ensure independent work, and help identify candidates who may warrant additional academic support upon enrollment. However, some candidates may feel intimidated or struggle with on-the-spot tasks, which could overshadow their other qualifications. It could also add time, cost, and complexity related to the design and evaluation of the task.

Data analysis followed reflexive thematic analysis approach [9,11] of individual brainstorming outputs, group-generated themes, creative reflection worksheets, and facilitator note identified three overarching areas of focus: interview format and delivery; AI integration and compliance; and admission criteria and evaluation. Within these, subthemes captured a range of perspectives including approaches to in-person and online interviews, supervised assessment, guidelines and disclosures, screening and awareness, detection and monitoring tools, and holistic evaluation strategies. In some cases, participants offered suggestions for strategies already used in the admissions process (noted by italics in Table 1); however, these suggestions often offered new perspectives or nuanced ideas for consideration.

**Table 1:** Key themes, sub-themes, and example brainstormed strategies identified through an admissions DT workshop.

Theme	Sub-Theme	Example Strategies
**Interview Format & Delivery**	In-Person & Online Interviews	· *Continue MMIs and verbal assessments* · Live assessments: written, verbal, math, group tasks · Continue live interviews online
	Supervised Assessments	· Observe students in real time · Live assessments: written, verbal, math, group tasks
	MMI Question Design & Innovation	· Chunk questions into smaller parts · Add a group-based station · *Introduce new questions each cycle* · Regularly restructure interviews
	Interview Oversight	· Form a dedicated technology committee for oversight and implementation
**AI Integration & Compliance**	Detection & Monitoring Tools	· Use tools to block or detect GenAI use · *Observe the candidate and their interview environment*
	Guidelines & Disclosures	· *Develop GenAI usage guidance for candidates* · Include GenAI disclosure in MMIs · Identify red and green flags in responses
	Screening & Awareness	· Use GenAI for initial candidate screening · Generate GenAI responses to raise awareness · Showcase student life via social media
**Admission Criteria & Evaluation**	Holistic Evaluation Approaches	· *Revisit criteria to expand or revise indicators and constructs of interest* · *Consider science/English language arts GPAs*
	Writing & Communication Samples	· Remove prewritten submissions (e.g., LOIs, essays) and replace with real-time submissions · Incorporate prior communication samples
	Experience & Personal Markers	· *Highlight unique experiences (e.g., wellness inventory, publications, work experience)*

NOTE: *Italicized* strategies are utilized in current admissions process.

Post-workshop survey data (n = 15, 94% response rate) are presented as descriptive indicators of participant experience. Participants generally reported positive perceptions of the workshop. They strongly agreed that the workshop encouraged creative problem solving (M = 4.7, SD = 0.5), helped them to identify potential strategies (4.8 ± 0.4), and enhanced the quality of ideas generated (M = 4.9, SD = 0.4). Most strongly agreed that the workshop was a good use of their time (Mean = 4.4, SD = 0.5) and that the results will be useful to the School (M = 4.8, SD = 0.4). Open-text responses emphasized the value of engaging diverse perspectives and highlighted the workshop as a space for structured dialogue. One participant described the process as engaging and effective for generating new ideas and meaningful discussion. Another participant suggested expanding the use of DT for future initiatives and across other committees or working groups. Suggestions for improvement included allowing more time for idea development and in one case, rotating group membership to further broaden perspectives and increase opportunities for cross-group interaction. These findings should be interpreted cautiously given the small sample and the potential for response and social desirability bias.

Additional reflections emerged during member-checking discussions with admission committee members, who offered additional strategies and raised several critical points for consideration. For example, one committee member suggested generating an MMI response from GenAI and presenting it to a candidate for critique. In addition, committee members challenged the utility and value of changing the admissions process (e.g., *Would we do anything different in our decision-making process?; How might we leverage admissions data in the curriculum once candidates enroll?; Can we obtain feedback from candidates?*)

Overall, these results demonstrate the range and diversity of ideas and perspectives elicited through a DT approach, highlighting its potential utility as a structured method for collaborative exploration and problem reframing in complex institutional contexts. However, no strategies were implemented or evaluated at the time of this writing, and the findings do not address feasibility, effectiveness, or impact.

## Discussion

This study illustrates how a DT approach can be used to explore complex and evolving challenges in student selection, including the advent of GenAI and sunsetting of standardized admissions tests. Specifically, we used a 3-phase DT approach to engage students, recruiters, faculty, and administrators in a process aimed at elucidating challenges and identifying potential solutions. The findings show how this process supported the articulation of key issues in contemporary admissions practices and enabled the development of a range of context-specific ideas, such as the use of GenAI, interview format (e.g., in-person/remote), and design of MMI stations.

Given the exploratory scope of this work, the findings do not demonstrate effectiveness or implementation. Instead, they provide insight into how stakeholders engaged with emerging issues. For example, the workshop highlighted differing perspectives on the role of GenAI in application materials and candidate evaluation. This reflects a broader shift in the literature, which points to increasing use of GenAI in admissions-related tasks. While the long-term impact of GenAI on student selection remains to be seen, scholars have quickly tested and critiqued its capabilities – with some demonstrating its ability to efficiently generate plausible college admissions essays [1,10]. Research suggests that applicants are increasingly likely to utilize GenAI, with one study citing a significant increase in its estimated use on medical school essays from 2021 to 2024 [2]. Further, Smith and colleagues reported that most medical residency applicants either used or anticipated using GenAI for assistance in developing their personal statement [12]. Through the DT workshop, our participants wrestled with the potential role of GenAI for applications and its implications for how we might evaluate candidates. We ideated potential strategies, such as removing pre-written components of the application and adding real-time writing assessments during the interview process, which may or may not be feasible for others, and we encourage contextualized exploration of localized challenges for further exploration of these issues.

Real-time interviews can provide critical insight into a candidate’s suitability for a health professions program; however, the structure of those interviews can vary widely. In-person interviews and remote interviews both provide benefits and drawbacks, requiring schools to determine an approach best suited to their needs. Remote interviews remove several barriers for candidates, including the cost of travel, reduction of time, and more flexibility [7]. This can increase the number of candidates who are able to interview at a program. Based on our experiences, many interviewers also prefer remote interviews because of the flexibility in location and reduced commitment time of participating in remote interviews versus in person. However, in person interviews remove the concern of candidates using GenAI during the interview, having others present during the interview process, or recording the interview questions, overall assuring the integrity of the process. To ensure candidates were aware that the use of GenAI was not permitted during the interview process, we developed usage guidelines to share with the candidates. We also reminded them of this prior to their interview in a group setting and had a short reminder added to all the MMI scenarios posted for candidates to consider.

Similarly, the design of individual MMI stations can vary significantly, affording schools the flexibility to focus on constructs best aligned with their values and priorities [3]. For example, an MMI station may be situational, asking candidates to consider and respond to a hypothetical scenario, or behavioral, asking candidates to interact with an actor who plays a specific role [4]. Stations may also be individual, involving a one-on-one interaction with the interviewer, or collaborative, involving work with other candidates or actors to complete a task or solve a problem [12]. Further, numerous constructs can be assessed in an MMI (e.g., empathy, ethical reasoning, critical thinking, self-awareness). This flexibility offers space for schools to think creatively about how to best design and leverage the MMI for decision-making in admissions. Our workshop resulted in suggestions for rethinking our MMI structure (e.g., adding a group task, making a station task-focused) and, as reflected in our member-checking process, schools should align the design and focus of their MMI with curricular priorities [4]. Further, all changes should be evaluated in the context of local policies and laws, such as the American Disabilities Act, with university resources consulted as needed (e.g., ADA university officers).

Beyond the specific ideas generated by the workshop, this study reflects the utility of DT for tackling complex challenges in a structured and efficient manner. This work contributes to a small, yet growing, body of literature in health professions education that leverages DT for problem solving, and the first known study to do so for student selection [13]. Our workshop was user-centered, focused on the needs and experiences of faculty, students and staff dedicated to the admissions process. It encouraged a deeper understanding of the problems faced by users and promoted brainstorming to generate a wide range of ideas. Our participants found the process useful and meaningful, suggesting that this approach may have utility for other types of educational challenges. By applying DT to the admissions process, we believe schools can create a user-centered, efficient, and effective system that meets the needs of prospective students and institutional constituents.

These examples illustrate how institutions might begin to examine similar challenges within their own contexts. In addition to the ideas generated, this study provides a practice-based example of DT as a structured approach to collaborative problem solving in health professions education. The workshop facilitated dialogue across stakeholder groups, brought forward diverse perspectives, and supported the generation of multiple potential strategies. Participants’ feedback indicated the process was engaging and useful for idea generation, however these perceptions should be interpreted with caution given the small sample size and reliance on self-reported data.

No interventions were implemented or evaluated as part of this study, and conclusions about feasibility, effectiveness, or scalability cannot be drawn. Instead, this work represents an early-stage exploration that may inform future cycles of prototyping and evaluation. Further research is needed to examine how ideas generated using DT processes can be translated into practice and assessed for their impact on admissions outcomes.

### Limitations and future research

There were several limitations that should be considered when interpreting this work. First, the workshop focused on a small purposively selected group of participants from a single institution, which limits generalizability of the insights generated. Second, the work focused on pharmacy education, which faces unique admissions challenges that may not be applicable in other health professions (e.g., retirement of the PCAT). As such, the results from this study should not be extrapolated to assume that all health professions and health professions schools experience similar challenges in student selection. Third, the work focused on the early, generative phases of DT process. No strategies were implemented or evaluated, and the findings do not provide evidence regarding feasibility, effectiveness, or impact. Instead, they reflect ideas and perspectives developed within a structured workshop setting. While multiple data sources were used to support a comprehensive view of the process, the analysis was intended to synthesize emergent themes rather than develop or test a theory.

This work provides a foundation for future research in two key areas. First, additional studies are needed to examine how DT can be applied across different educational and organizational contexts, including its role in supporting stakeholder engagement, idea development, and subsequent stages of prototyping and evaluation. Second, additional research should evaluate the outcomes of strategies generated through such processes. This may include testing selected ideas, conducting multi-site or longitudinal studies, and using more robust mixed-methods or experimental designs to evaluate impact.

In the context of student selection, future work should explore whether the ideas identified here are applicable to health professions programs and consider how the DT framework can further support the identification and design of solutions to complex problems within student selection. Specifically, future research should explore the implications of GenAI and other emerging technologies for student selection, including the use of GenAI by candidates and by admissions officers. The capabilities of GenAI are advancing rapidly, making it critical for health professions programs to respond quickly. Additional research is also needed to develop and evaluate admissions criteria that appropriately capture institutionally relevant characteristics; and explore new approaches to assessing these characteristics (e.g., new interview models).

Although currently outside the scope of this study, evaluating the effectiveness of any implemented solutions would be a critical next step for further demonstrating the utility of DT.

## Conclusion

DT offers a systematic, iterative methodology for exploring the shifting complexities of admissions. By emphasizing collaboration and creative problem-solving, it enables teams to generate and refine innovative ideas through structured ideation and prototyping. This study illustrates how such an approach can be used to explore challenges, surface diverse perspectives, and generate context-specific strategies. While effectiveness was not assessed, this work highlights the potential of DT as a foundation for future development and evaluation of admissions practices.

## Abbreviations

DT: Design Thinking
PharmD: Doctor of Pharmacy
