# Supplementary material for: Leveraging design thinking to tackle contemporary admissions challenges in health professions education
Source: PLoS One. 2026 Jun 23;21(6):e0352192. doi: 10.1371/journal.pone.0352192 (PMC13289911; doi:10.1371/journal.pone.0352192)
Supplement: Attachment — Submitted filename: Response to Reviewers.docx [file pone.0352192.s002.docx]

**Response to Reviewers**

Thank you for your thoughtful comments and constructive suggestions. We have carefully considered each of your points and have revised the manuscript accordingly. Below, we provide a point-by-point response outlining how we have addressed your questions and comments, and the corresponding changes have been made in the revised version of the manuscript.

1. ***Please provide additional details regarding participant consent. In the ethics statement in the Methods and online submission information, please ensure that you have specified (1) whether consent was informed and (2) what type you obtained (for instance, written or verbal, and if verbal, how it was documented and witnessed). If your study included minors, state whether you obtained consent from parents or guardians. If the need for consent was waived by the ethics committee, please include this information.* *If you are reporting a retrospective study of medical records or archived samples, please ensure that you have discussed whether all data were fully anonymized before you accessed them and/or whether the IRB or ethics committee waived the requirement for informed consent. If patients provided informed written consent to have data from their medical records used in research, please include this information.***

Additional details have been added about consent and anonymity in the IRB statement	of the methods. This study was determined to be not human subjects research by the	University of North Carolina at Chapel Hill Institutional Review Board (IRB Number: 24-	3149). Informed consent was not required by the IRB and was not obtained. All data	were collected anonymously.

No minors were included in this research.

**Reviewer #1:**

1. ***I would like to see more detail about the workshop design and strategic choices made to limit the scope of discussion to the three launch points as they relate to expressed concerns or reactions to policy or procedure changes.***

Additional detail added about inspiration for the launching points (e.g., recent experiences and pain points, such as the loss of the PCAT as a reliable source of candidate comparison).

2. ***While the introduction summarizes many challenges that provide background for the workshop, the authors should better define the specific topics covered by each launch point (page 6-7). I presume the term "Technology" is specific to generative AI, though strategic use of technology may extend to predictive AI based on curricular outcomes which should serve to inform admissions standards and expectations. Should virtual conferencing technology be included or considered? would the admissions committee consider recorded video interview technology (Kira Talent) or situational judgment assessments (Acuity Insights Casper/Duet)?***

Additional detail about the launching points has been added, including the problem statement that described aspects of focus for the workshop.

**Each launch point should be associated with a specific problem statement (challenge) that contextualizes each launch point. Launch point 2 (how to reliably compare candidates) is an ever-present challenge, so the authors should explicitly state that this point's urgency is due to the discontinuation of the PCAT or the concern of critical thinking deficiencies or math skills for entering students. The authors suggest that "statements" were crafted to define each launching point, so it may help the reader to know what those prompts were in their final form.**

The statements that accompanied the launching points have been added:

- Statement 1: Candidates are utilizing various technologies during the application process (e.g., application, interview). How might we ensure that candidates are using technology in fair and ethical ways?
- Statement 2: Candidates have varied levels of experience, knowledge, and preparedness for pharmacy school. How might we reliably compare candidates in a post-PCAT world?
- Statement 3: Our admissions process utilizes various technologies for the admissions process (e.g., online application, Kira talent) How might we strategically utilize current and emerging technologies (e.g., GenAI) in the admissions process?

3. **The authors should be more explicit in describing the constraints on the generated ideas (cost, geography, and equity, line 143). Examples of pros/cons of example ideas would be helpful to underscore how these constraints helped in shortlisting suggestions for the admissions committee's consideration.**

Additional information about the constraints have been added, along with examples to help illustrate these concepts for readers. Per your comment below, we have included disability as an equity example.

4. ***The authors should comment about potentially disadvantaging candidates with disabilities that could affect an assessment under time pressure. For example, non-native English speaking/international applicants may find AI assistance helpful to better understand question prompts or articulate their opinions when writing essays. How will these suggestions be evaluated when it comes to fair comparison of candidate competencies and technical abilities? Are there protocols in place for those with dyslexia or dyscalculia, or those with impaired hearing who may require assistance?***

This is a great point and we appreciate your recommendation. All changes would be evaluated in the context of ADA and disability (we consider this part of equity, as noted above). We’ve added text to the paper to bring this forward.

5. ***Were ADA university officers consulted before the shortlist suggestions were presented to the admissions committee? After they were presented?***

ADA officers were not consulted in this workshop, but we appreciate this suggestion	and have noted it in the discussion as something for others to consider.

6. ***I would like to see more information about the tenure of those participating in the DT workshop aside from their specific roles at the time the workshop was held (page 6 line 118). Specifically, what was the range of years serving on the admissions committee, as interviewers, or as file screeners. How many tenured faculty were included, or was there a balance between research-focused and practice-focused faculty? What proportions of the entire committee roster, admissions office, or student ambassador corps were represented?***

Additional details about participants was added.

7. **Did the authors serve as facilitators or participants in brainstorming?**

Added to the methods that two authors served as facilitators and one as a participant.

8. ***I also think it is important to describe the race/ethnicity, education/expertise, gender (identity), disability, and language diversity of the assembled workshop participants to show the diversity of the group. This includes knowing the year each student ambassador has been enrolled at the school and/or served as an admissions volunteer ambassador (P2, P3, P4, graduate). Are alumni involved with candidate selection?***

Demographic data was not collected from participants, so we’re unable to provide most of this information. We have noted the year of the student ambassadors and the numbers of participants that were alumni.

9. ***Finally, were there specific changes that were considered in screening, interview, or final file assessment criteria or rubrics as a result of the workshop? How did each suggestion affect the three stage file review process for entering students (academic review, comprehensive review, and post-interview decision? Is there a way to highlight suggestions within the context of the three-stage admissions process? How do these changes align with the intended mission and core values of the institution? As such, the conclusion of the results section seems unsatisfying ("challenged the utility and value of changing the process").***

Describing changes made to our admissions process is beyond the scope of this work. However, we did implement a live writing assessment as part of this year’s admissions cycle based on the outcomes of the design thinking session. Other changes based on recommendations received may be implemented in the upcoming admission cycle when there is more time to plan and implement the change.

10. ***I appreciate the optimism of the authors to call upon their peers to consider similar approaches to design appropriate solutions. Many of the suggestions made line up with solutions suggested by other admissions committees who did NOT go through a DT process.* *I would appreciate more insight into the rationale, implementation, and assessment of each suggestion (for example, will the live assessments occur during the MMI period?). What happens if a candidate is found to be overreliant on AI tools (we had similar concerns about plagiarism over 10 years ago)? Tie the specific suggestions to the launch points and/or the context/challenge the launch point addresses.***

This manuscript is focused on describing the implementation and evaluation of a design thinking workshop focused on admissions challenges. The subsequent work of selecting, implementing and assessing changes to the admissions process is beyond the scope of this work.

11. ***I anticipate many admissions professionals may go directly to the suggestions mentioned in the table and try implementing them without going through a DT process. I would be interested in how to fairly present the suggestions and directly comment on the barriers to implementation or their assessment, including the nature of human oversight over results through AI tools.***

We appreciate this challenge. However, the table is intended to reflect the utility of a DT workshop for generating strategies that may be useful for admissions. Detailing barriers to each strategies is or theme is beyond the scope of the work. To help emphasize this point, we have revised the title.

**Minor corrections/issues:**

12. **Page 16, Line 306: "Data Chowuration"**

Addressed.

**Reviewer #2:**

**First, in discussing the qualitative methodology beginning on Line 155, additional clarifications should be provided. I would start with Braun and Clark's work, and elucidate the chosen process for the qualitative coding of the data.**

Clarifications regarding methodology were added including the process for qualitative coding of the data.

13. **Second, more explanation is needed to understand where the statistic 90.5% actually comes from when citing intercoder reliability. How was this measured since it is not presented as a Cohen's kappa?**

Further explanation was added regarding the process for calculating intercoder reliability.

14. **Beginning on Line 190, it is unclear exactly which data are actually coded in the analysis. Is the analysis referring to the 76 initial ideas, or the 43 selected for further discussion? More information on this part of the process is necessary. Providing an n = #, next to each of the main themes, and the sub themes in the paragraph starting on Line 190 would be helpful to better contextualize the data. For example, was one of the 3 themes completely dominant with 40 responses (of 43? 76? other?), or were the three themes more equally distributed in the number of mentions they received? What ideas fell outside of the 3 main themes? What other possible themes were suggested during the coding process? Again, it would be helpful to know what data were actually coded for this reporting. This part of the paper will benefit from a revision of how the qualitative data are presented, and starting with Braun and Clark's model and presentation will be very beneficial here.**

Further clarification regarding data analysis was added. Examples of suggested themes that fell outside of the three main themes were provided.

15. **I would also revise the lines 201-202, instead of stating that participants "rated the workshop highly", the writing should reflect the actual question asked (which was a strongly agree to strongly disagree question). For example, participants strongly agreed on X, M= 4.8, SD = 0.4. The authors could also choose to represent this data in a different format, such as 95% of participants strongly agreed (or what was the next one at a rating of 4?) on X statement, M = 4.8, SD = 0.4. Also, on line 152 the authors should identify each of the likert scale points (what does a 2, 3 or 4 mean? slightly disagree? moderately? is selecting a 3 meaning "neither disagree or agree?" More specificity would be helpful.**

Additional specificity regarding the post-evaluation survey was included.

16. **Given that we don’t know which of the data types were coded in the qualitative reporting section, I am assuming that there is no data presented from the open-ended post workshop question from the survey (with the other likert scale items). The paper presents its purpose as demonstrating how DT can support admissions challenges and collaborative brainstorming, but then doesn't say anything about how participants experienced the process of DT through the workshop. Revisiting those 15 open-ended responses (if they got that many to write in something), would be really revealing as to how the process of participating in the workshop went. That qualitative data can add nuance to the likert data. If no one responded to the open-ended question, the authors could also state that.**

Additional information regarding the responses to the open-ended question on the post-evaluation survey was added.

**Finally, there are a few small editorial fixes here:**

**17. Line 93: Add a comma after "work"**

Addressed.

**18. Line 132: Add a colon after "3"**

Addressed.

**19. Line 186 Add an s to the word "candidate**"

Addressed.
