# Supplementary material for: Leveraging design thinking to tackle contemporary admissions challenges in health professions education
Source: PLoS One. 2026 Jun 23;21(6):e0352192. doi: 10.1371/journal.pone.0352192 (PMC13289911; doi:10.1371/journal.pone.0352192)
Supplement: Attachment — Submitted filename: Response to Academic Editor comments.docx [file pone.0352192.s003.docx]

**Response to Academic Editor comments**

1. *The primary concern relates to the nature of the evidence generated. The study is based on a single, short-duration workshop involving a small, purposively selected group of participants from one institution (n=15).*

We acknowledge this limitation and have addressed it throughout the manuscript by clarifying the exploratory nature of the study, the purposive sampling approach, and the limited generalizability associated with a single, small sample, short-duration workshop conducted at one institution.

2. *The outcomes reported are largely descriptive and exploratory, consisting of generated ideas, thematic categorizations, and participants’ perceptions of the workshop experience. While these findings are informative, they do not provide empirical evidence regarding the effectiveness, feasibility, or impact of the proposed strategies.*

We have clarified throughout the manuscript that the purpose of this work was to inform early-stage strategy development and gather stakeholder insights, rather than to assess intervention outcomes or establish evidence of impact.

3. *In particular, the quantitative component is limited to self-reported perceptions measured via Likert-scale responses, which are inherently subject to response and social desirability biases. The qualitative component, although appropriate in principle, is based on heterogeneous data sources (brainstorming outputs, facilitator notes, and reflection materials), and the analytical approach, while improved, remains relatively limited in methodological depth.*

We have further acknowledged these methodological limitations throughout the manuscript, including the reliance on self-reported perceptions and the use of multiple qualitative sources, and clarified the scope and interpretive boundaries of the analysis presented.

4. *A further important limitation is the absence of any evaluation of implemented interventions. Although the manuscript discusses potential strategies and prototypes generated through the DT process, it does not assess whether these strategies were adopted, nor whether they resulted in measurable improvements in admissions processes or outcomes. As such, the manuscript primarily demonstrates that design thinking can facilitate idea generation, rather than providing evidence of its effectiveness as an intervention.*

We have acknowledged throughout the manuscript that the study did not include implementation or outcome evaluation of the proposed strategies and clarified that the work was intended to demonstrate the utility of the design thinking process for stakeholder engagement and idea generation.

5. *More broadly, the manuscript would benefit from clearer conceptual positioning. Clarifying this positioning is essential to ensure alignment between the study design, the claims made, and the expectations of the journal.*

We have clarified the conceptual positioning of the study throughout the manuscript to better align the study aims, design, and conclusions with the exploratory scope of the work.

6. *Finally, while the conclusions are generally reasonable, they should be more explicitly grounded in the exploratory nature of the data. Statements regarding the value and utility of design thinking should be appropriately tempered to reflect the absence of outcome-based evidence.*

We have revised the conclusions throughout the manuscript to more clearly reflect the exploratory nature of the findings and to ensure that the statements regarding the value and utility of design thinking are appropriately contextualized within the scope of the evidence presented.

7. *In summary, your manuscript addresses an important topic and presents a well-described and potentially useful approach to addressing complex admissions challenges. However, substantial revisions are required to strengthen the conceptual framing, appropriately calibrate the interpretation of findings, and clarify the contribution of the study.*

We have made substantial revisions throughout the manuscript to strengthen the conceptual framing, better align the interpretation of the findings with the exploratory nature of the study, and clarify the overall contribution of the work.
