# Supplementary material for: Leveraging design thinking to tackle contemporary admissions challenges in health professions education
Source: PLoS One. 2026 Jun 23;21(6):e0352192. doi: 10.1371/journal.pone.0352192 (PMC13289911; doi:10.1371/journal.pone.0352192)
Supplement: Peer review history

# Peer review: Decision Letter 0

**DOI:** 10.1371/journal.pone.0352192.r001

**Authors:** Kendra Oliver

16 Jan 2026

PONE-D-25-54674

Leveraging design thinking to tackle contemporary admissions challenges in health professions education

PLOS One

Dear Dr. McLaughlin,

Thank you for submitting your manuscript to PLOS ONE. After careful consideration, we feel that it has merit but does not fully meet PLOS ONE’s publication criteria as it currently stands. Therefore, we invite you to submit a revised version of the manuscript that addresses the points raised during the review process.

Please submit your revised manuscript by Mar 02 2026 11:59PM. If you will need more time than this to complete your revisions, please reply to this message or contact the journal office at plosone@plos.org. When you're ready to submit your revision, log on to https://www.editorialmanager.com/pone/ and select the 'Submissions Needing Revision' folder to locate your manuscript file.

Please include the following items when submitting your revised manuscript:

- A letter that responds to each point raised by the academic editor and reviewer(s). You should upload this letter as a separate file labeled 'Response to Reviewers'.
- A marked-up copy of your manuscript that highlights changes made to the original version. You should upload this as a separate file labeled 'Revised Manuscript with Track Changes'.
- An unmarked version of your revised paper without tracked changes. You should upload this as a separate file labeled 'Manuscript'.

-->If you would like to make changes to your financial disclosure, please include your updated statement in your cover letter. Guidelines for resubmitting your figure files are available below the reviewer comments at the end of this letter.

If applicable, we recommend that you deposit your laboratory protocols in protocols.io to enhance the reproducibility of your results. Protocols.io assigns your protocol its own identifier (DOI) so that it can be cited independently in the future. For instructions see: https://journals.plos.org/plosone/s/submission-guidelines#loc-laboratory-protocols. Additionally, PLOS ONE offers an option for publishing peer-reviewed Lab Protocol articles, which describe protocols hosted on protocols.io. Read more information on sharing protocols at https://plos.org/protocols?utm_medium=editorial-email&utm_source=authorletters&utm_campaign=protocols.

We look forward to receiving your revised manuscript.

Kind regards,

Kendra Helen Oliver, Ph.D.

Academic Editor

PLOS One

Journal requirements:

When submitting your revision, we need you to address these additional requirements.

1. Please ensure that your manuscript meets PLOS ONE's style requirements, including those for file naming. The PLOS ONE style templates can be found at

https://journals.plos.org/plosone/s/file?id=wjVg/PLOSOne_formatting_sample_main_body.pdf and

https://journals.plos.org/plosone/s/file?id=ba62/PLOSOne_formatting_sample_title_authors_affiliations.pdf

2. Please provide additional details regarding participant consent. In the ethics statement in the Methods and online submission information, please ensure that you have specified (1) whether consent was informed and (2) what type you obtained (for instance, written or verbal, and if verbal, how it was documented and witnessed). If your study included minors, state whether you obtained consent from parents or guardians. If the need for consent was waived by the ethics committee, please include this information.

If you are reporting a retrospective study of medical records or archived samples, please ensure that you have discussed whether all data were fully anonymized before you accessed them and/or whether the IRB or ethics committee waived the requirement for informed consent. If patients provided informed written consent to have data from their medical records used in research, please include this information.

If the reviewer comments include a recommendation to cite specific previously published works, please review and evaluate these publications to determine whether they are relevant and should be cited. There is no requirement to cite these works unless the editor has indicated otherwise.

Please review your reference list to ensure that it is complete and correct. If you have cited papers that have been retracted, please include the rationale for doing so in the manuscript text, or remove these references and replace them with relevant current references. Any changes to the reference list should be mentioned in the rebuttal letter that accompanies your revised manuscript. If you need to cite a retracted article, indicate the article’s retracted status in the References list and also include a citation and full reference for the retraction notice.

[Note: HTML markup is below. Please do not edit.]

Reviewers' comments:

Reviewer's Responses to Questions

**Comments to the Author**

1. Is the manuscript technically sound, and do the data support the conclusions?

The manuscript must describe a technically sound piece of scientific research with data that supports the conclusions. Experiments must have been conducted rigorously, with appropriate controls, replication, and sample sizes. The conclusions must be drawn appropriately based on the data presented.

Reviewer #1: Yes

Reviewer #2: Yes

**********

2. Has the statistical analysis been performed appropriately and rigorously?

Reviewer #1: Yes

Reviewer #2: No

**********

3. Have the authors made all data underlying the findings in their manuscript fully available?

The PLOS Data policy requires authors to make all data underlying the findings described in their manuscript fully available without restriction, with rare exception (please refer to the Data Availability Statement in the manuscript PDF file). The data should be provided as part of the manuscript or its supporting information, or deposited to a public repository. For example, in addition to summary statistics, the data points behind means, medians and variance measures should be available. If there are restrictions on publicly sharing data—e.g. participant privacy or use of data from a third party—those must be specified.

Reviewer #1: Yes

Reviewer #2: Yes

**********

4. Is the manuscript presented in an intelligible fashion and written in standard English?

PLOS ONE does not copyedit accepted manuscripts, so the language in submitted articles must be clear, correct, and unambiguous. Any typographical or grammatical errors should be corrected at revision, so please note any specific errors here.

Reviewer #1: Yes

Reviewer #2: Yes

**********

5. Review Comments to the Author

Please use the space provided to explain your answers to the questions above. You may also include additional comments for the author, including concerns about dual publication, research ethics, or publication ethics. (Please upload your review as an attachment if it exceeds 20,000 characters)

Reviewer #1: I appreciate the authors' approach to develop collaborative suggestions through design thinking to address concerns in student selection. The paper is focused on a structured process of brainstorming and shortlisting suggestions so adapt an admissions committee process. Overall, the summary is well-written, and I am hopeful that peers in admissions, recruitment, and enrollment will consider using a similar approach to thoughtfully tweak admissions processes. However, I would like to see more detail about the workshop design and strategic choices made to limit the scope of discussion to the three launch points as they relate to expressed concerns or reactions to policy or procedure changes.

While the introduction summarizes many challenges that provide background for the workshop, the authors should better define the specific topics covered by each launch point (page 6-7). I presume the term "Technology" is specific to generative AI, though strategic use of technology may extend to predictive AI based on curricular outcomes which should serve to inform admissions standards and expectations. Should virtual conferencing technology be included or considered? would the admissions committee consider recorded video interview technology (Kira Talent) or situational judgment assessments (Acuity Insights Casper/Duet)? Each launch point should be associated with a specific problem statement (challenge) that contextualizes each launch point. Launch point 2 (how to reliably compare candidates) is an ever-present challenge, so the authors should explicitly state that this point's urgency is due to the discontinuation of the PCAT or the concern of critical thinking deficiencies or math skills for entering students. The authors suggest that "statements" were crafted to define each launching point, so it may help the reader to know what those prompts were in their final form.

The authors should be more explicit in describing the constraints on the generated ideas (cost, geography, and equity, line 143). Examples of pros/cons of example ideas would be helpful to underscore how these constraints helped in shortlisting suggestions for the admissions committee's consideration.

The authors should comment about potentially disadvantaging candidates with disabilities that could affect an assessment under time pressure. For example, non-native English speaking/international applicants may find AI assistance helpful to better understand question prompts or articulate their opinions when writing essays. How will these suggestions be evaluated when it comes to fair comparison of candidate competencies and technical abilities? Are there protocols in place for those with dyslexia or dyscalculia, or those with impaired hearing who may require assistance? Were ADA university officers consulted before the shortlist suggestions were presented to the admissions committee? After they were presented?

I would like to see more information about the tenure of those participating in the DT workshop aside from their specific roles at the time the workshop was held (page 6 line 118). Specifically, what was the range of years serving on the admissions committee, as interviewers, or as file screeners. How many tenured faculty were included, or was there a balance between research-focused and practice-focused faculty? What proportions of the entire committee roster, admissions office, or student ambassador corps were represented? Did the authors serve as facilitators or participants in brainstorming?

I also think it is important to describe the race/ethnicity, education/expertise, gender (identity), disability, and language diversity of the assembled workshop participants to show the diversity of the group. This includes knowing the year each student ambassador has been enrolled at the school and/or served as an admissions volunteer ambassador (P2, P3, P4, graduate). Are alumni involved with candidate selection?

Finally, were there specific changes that were considered in screening, interview, or final file assessment criteria or rubrics as a result of the workshop? How did each suggestion affect the three stage file review process for entering students (academic review, comprehensive review, and post-interview decision? Is there a way to highlight suggestions within the context of the three-stage admissions process? How do these changes align with the intended mission and core values of the institution? As such, the conclusion of the results section seems unsatisfying ("challenged the utility and value of changing the process").

I appreciate the optimism of the authors to call upon their peers to consider similar approaches to design appropriate solutions. Many of the suggestions made line up with solutions suggested by other admissions committees who did NOT go through a DT process. I would appreciate more insight into the rationale, implementation, and assessment of each suggestion (for example, will the live assessments occur during the MMI period?). What happens if a candidate is found to be overreliant on AI tools (we had similar concerns about plagiarism over 10 years ago)? Tie the specific suggestions to the launch points and/or the context/challenge the launch point addresses.

I anticipate many admissions professionals may go directly to the suggestions mentioned in the table and try implementing them without going through a DT process. I would be interested in how to fairly present the suggestions and directly comment on the barriers to implementation or their assessment, including the nature of human oversight over results through AI tools.

Minor corrections/issues:

Page 16, Line 306: "Data Chowuration"

Reviewer #2: Thank you for this contribution to the field.

As someone who has worked on designing rubrics for holistic admissions in the biological and biomedical sciences, I see the need for the work this study presents. Overall, it is well done, but has a few areas in need of improvement/clarity.

First, in discussing the qualitative methodology beginning on Line 155, additional clarifications should be provided. I would start with Braun and Clark's work, and elucidate the chosen process for the qualitative coding of the data. Second, more explanation is needed to understand where the statistic 90.5% actually comes from when citing intercoder reliability. How was this measured since it is not presented as a Cohen's kappa?

Beginning on Line 190, it is unclear exactly which data are actually coded in the analysis. Is the analysis referring to the 76 initial ideas, or the 43 selected for further discussion? More information on this part of the process is necessary. Providing an n = #, next to each of the main themes, and the sub themes in the paragraph starting on Line 190 would be helpful to better contextualize the data. For example, was one of the 3 themes completely dominant with 40 responses (of 43? 76? other?), or were the three themes more equally distributed in the number of mentions they received? What ideas fell outside of the 3 main themes? What other possible themes were suggested during the coding process? Again, it would be helpful to know what data were actually coded for this reporting. This part of the paper will benefit from a revision of how the qualitative data are presented, and starting with Braun and Clark's model and presentation will be very beneficial here.

I would also revise the lines 201-202, instead of stating that participants "rated the workshop highly", the writing should reflect the actual question asked (which was a strongly agree to strongly disagree question). For example, participants strongly agreed on X, M= 4.8, SD = 0.4. The authors could also choose to represent this data in a different format, such as 95% of participants strongly agreed (or what was the next one at a rating of 4?) on X statement, M = 4.8, SD = 0.4. Also, on line 152 the authors should identify each of the likert scale points (what does a 2, 3 or 4 mean? slightly disagree? moderately? is selecting a 3 meaning "neither disagree or agree?" More specificity would be helpful.

Given that we dont know which of the data types were coded in the qualitative reporting section, I am assuming that there is no data presented from the open-ended post workshop question from the survey (with the other likert scale items). The paper presents its purpose as demonstrating how DT can support admissions challenges and collaborative brainstorming, but then doesn't say anything about how participants experienced the process of DT through the workshop. Revisiting those 15 open-ended responses (if they got that many to write in something), would be really revealing as to how the process of participating in the workshop went. That qualitative data can add nuance to the likert data. if no one responded to the open ended question, the authors could also state that.

Finally, there are a few small editorial fixes here:

1. Line 93: Add a comma after "work"

2. Line 132: Add a colon after "3"

3. Line 186 Add an s to the word "candidate"

I think this paper has great merit and once the authors address some of the qualitative data concerns, I hope to revisit this work! Thank you!

**********

6. PLOS authors have the option to publish the peer review history of their article (what does this mean?). If published, this will include your full peer review and any attached files.

If you choose “no”, your identity will remain anonymous but your review may still be made public.

**Do you want your identity to be public for this peer review?**  For information about this choice, including consent withdrawal, please see our Privacy Policy.

Reviewer #1: **Yes:** Emil Chuck

Reviewer #2: No

**********

[NOTE: If reviewer comments were submitted as an attachment file, they will be attached to this email and accessible via the submission site. Please log into your account, locate the manuscript record, and check for the action link "View Attachments". If this link does not appear, there are no attachment files.]

To ensure your figures meet our technical requirements, please review our figure guidelines: https://journals.plos.org/plosone/s/figures

You may also use PLOS’s free figure tool, NAAS, to help you prepare publication quality figures: https://journals.plos.org/plosone/s/figures#loc-tools-for-figure-preparation.

NAAS will assess whether your figures meet our technical requirements by comparing each figure against our figure specifications.

# Author comment: Author response to Decision Letter 1

**DOI:** 10.1371/journal.pone.0352192.r002

25 Feb 2026

Thank you for your thoughtful comments and constructive suggestions. We have carefully considered each of your points and have revised the manuscript accordingly. Below, we provide a point-by-point response outlining how we have addressed your questions and comments, and the corresponding changes have been made in the revised version of the manuscript.

1. Please provide additional details regarding participant consent. In the ethics statement in the Methods and online submission information, please ensure that you have specified (1) whether consent was informed and (2) what type you obtained (for instance, written or verbal, and if verbal, how it was documented and witnessed). If your study included minors, state whether you obtained consent from parents or guardians. If the need for consent was waived by the ethics committee, please include this information. If you are reporting a retrospective study of medical records or archived samples, please ensure that you have discussed whether all data were fully anonymized before you accessed them and/or whether the IRB or ethics committee waived the requirement for informed consent. If patients provided informed written consent to have data from their medical records used in research, please include this information.

Additional details have been added about consent and anonymity in the IRB statement of the methods. This study was determined to be not human subjects research by the University of North Carolina at Chapel Hill Institutional Review Board (IRB Number: 24- 3149). Informed consent was not required by the IRB and was not obtained. All data were collected anonymously.

No minors were included in this research.

Reviewer #1:

1. I would like to see more detail about the workshop design and strategic choices made to limit the scope of discussion to the three launch points as they relate to expressed concerns or reactions to policy or procedure changes.

Additional detail added about inspiration for the launching points (e.g., recent experiences and pain points, such as the loss of the PCAT as a reliable source of candidate comparison).

2. While the introduction summarizes many challenges that provide background for the workshop, the authors should better define the specific topics covered by each launch point (page 6-7). I presume the term "Technology" is specific to generative AI, though strategic use of technology may extend to predictive AI based on curricular outcomes which should serve to inform admissions standards and expectations. Should virtual conferencing technology be included or considered? would the admissions committee consider recorded video interview technology (Kira Talent) or situational judgment assessments (Acuity Insights Casper/Duet)?

Additional detail about the launching points has been added, including the problem statement that described aspects of focus for the workshop.

Each launch point should be associated with a specific problem statement (challenge) that contextualizes each launch point. Launch point 2 (how to reliably compare candidates) is an ever-present challenge, so the authors should explicitly state that this point's urgency is due to the discontinuation of the PCAT or the concern of critical thinking deficiencies or math skills for entering students. The authors suggest that "statements" were crafted to define each launching point, so it may help the reader to know what those prompts were in their final form.

The statements that accompanied the launching points have been added:

• Statement 1: Candidates are utilizing various technologies during the application process (e.g., application, interview). How might we ensure that candidates are using technology in fair and ethical ways?

• Statement 2: Candidates have varied levels of experience, knowledge, and preparedness for pharmacy school. How might we reliably compare candidates in a post-PCAT world?

• Statement 3: Our admissions process utilizes various technologies for the admissions process (e.g., online application, Kira talent) How might we strategically utilize current and emerging technologies (e.g., GenAI) in the admissions process?

3. The authors should be more explicit in describing the constraints on the generated ideas (cost, geography, and equity, line 143). Examples of pros/cons of example ideas would be helpful to underscore how these constraints helped in shortlisting suggestions for the admissions committee's consideration.

Additional information about the constraints have been added, along with examples to help illustrate these concepts for readers. Per your comment below, we have included disability as an equity example.

4. The authors should comment about potentially disadvantaging candidates with disabilities that could affect an assessment under time pressure. For example, non-native English speaking/international applicants may find AI assistance helpful to better understand question prompts or articulate their opinions when writing essays. How will these suggestions be evaluated when it comes to fair comparison of candidate competencies and technical abilities? Are there protocols in place for those with dyslexia or dyscalculia, or those with impaired hearing who may require assistance?

This is a great point and we appreciate your recommendation. All changes would be evaluated in the context of ADA and disability (we consider this part of equity, as noted above). We’ve added text to the paper to bring this forward.

5. Were ADA university officers consulted before the shortlist suggestions were presented to the admissions committee? After they were presented?

ADA officers were not consulted in this workshop, but we appreciate this suggestion and have noted it in the discussion as something for others to consider.

6. I would like to see more information about the tenure of those participating in the DT workshop aside from their specific roles at the time the workshop was held (page 6 line 118). Specifically, what was the range of years serving on the admissions committee, as interviewers, or as file screeners. How many tenured faculty were included, or was there a balance between research-focused and practice-focused faculty? What proportions of the entire committee roster, admissions office, or student ambassador corps were represented?

Additional details about participants was added.

7. Did the authors serve as facilitators or participants in brainstorming?

Added to the methods that two authors served as facilitators and one as a participant.

8. I also think it is important to describe the race/ethnicity, education/expertise, gender (identity), disability, and language diversity of the assembled workshop participants to show the diversity of the group. This includes knowing the year each student ambassador has been enrolled at the school and/or served as an admissions volunteer ambassador (P2, P3, P4, graduate). Are alumni involved with candidate selection?

Demographic data was not collected from participants, so we’re unable to provide most of this information. We have noted the year of the student ambassadors and the numbers of participants that were alumni.

9. Finally, were there specific changes that were considered in screening, interview, or final file assessment criteria or rubrics as a result of the workshop? How did each suggestion affect the three stage file review process for entering students (academic review, comprehensive review, and post-interview decision? Is there a way to highlight suggestions within the context of the three-stage admissions process? How do these changes align with the intended mission and core values of the institution? As such, the conclusion of the results section seems unsatisfying ("challenged the utility and value of changing the process").

Describing changes made to our admissions process is beyond the scope of this work. However, we did implement a live writing assessment as part of this year’s admissions cycle based on the outcomes of the design thinking session. Other changes based on recommendations received may be implemented in the upcoming admission cycle when there is more time to plan and implement the change.

10. I appreciate the optimism of the authors to call upon their peers to consider similar approaches to design appropriate solutions. Many of the suggestions made line up with solutions suggested by other admissions committees who did NOT go through a DT process. I would appreciate more insight into the rationale, implementation, and assessment of each suggestion (for example, will the live assessments occur during the MMI period?). What happens if a candidate is found to be overreliant on AI tools (we had similar concerns about plagiarism over 10 years ago)? Tie the specific suggestions to the launch points and/or the context/challenge the launch point addresses.

This manuscript is focused on describing the implementation and evaluation of a design thinking workshop focused on admissions challenges. The subsequent work of selecting, implementing and assessing changes to the admissions process is beyond the scope of this work.

11. I anticipate many admissions professionals may go directly to the suggestions mentioned in the table and try implementing them without going through a DT process. I would be interested in how to fairly present the suggestions and directly comment on the barriers to implementation or their assessment, including the nature of human oversight over results through AI tools.

We appreciate this challenge. However, the table is intended to reflect the utility of a DT workshop for generating strategies that may be useful for admissions. Detailing barriers to each strategies is or theme is beyond the scope of the work. To help emphasize this point, we have revised the title.

Minor corrections/issues:

12. Page 16, Line 306: "Data Chowuration"

Addressed.

Reviewer #2:

First, in discussing the qualitative methodology beginning on Line 155, additional clarifications should be provided. I would start with Braun and Clark's work, and elucidate the chosen process for the qualitative coding of the data.

Clarifications regarding methodology were added including the process for qualitative coding of the data.

13. Second, more explanation is needed to understand where the statistic 90.5% actually comes from when citing intercoder reliability. How was this measured since it is not presented as a Cohen's kappa?

Further explanation was added regarding the process for calculating intercoder reliability.

14. Beginning on Line 190, it is unclear exactly which data are actually coded in the analysis. Is the analysis referring to the 76 initial ideas, or the 43 selected for further discussion? More information on this part of the process is necessary. Providing an n = #, next to each of the main themes, and the sub themes in the paragraph starting on Line 190 would be helpful to better contextualize the data. For example, was one of the 3 themes completely dominant with 40 responses (of 43? 76? other?), or were the three themes more equally distributed in the number of mentions they received? What ideas fell outside of the 3 main themes? What other possible themes were suggested during the coding process? Again, it would be helpful to know what data were actually coded for this reporting. This part of the paper will benefit from a revision of how the qualitative data are presented, and starting with Braun and Clark's model and presentation will be very beneficial here.

Further clarification regarding data analysis was added. Examples of suggested themes that fell outside of the three main themes were provided.

15. I would also revise the lines 201-202, instead of stating that participants "rated the workshop highly", the writing should reflect the actual question asked (which was a strongly agree to strongly disagree question). For example, participants strongly agreed on X, M= 4.8, SD = 0.4. The authors could also choose to represent this data in a different format, such as 95% of participants strongly agreed (or what was the next one at a rating of 4?) on X statement, M = 4.8, SD = 0.4. Also, on line 152 the authors should identify each of the likert scale points (what does a 2, 3 or 4 mean? slightly disagree? moderately? is selecting a 3 meaning "neither disagree or agree?" More specificity would be helpful.

Additional specificity regarding the post-evaluation survey was included.

16. Given that we don’t know which of the data types were coded in the qualitative reporting section, I am assuming that there is no data presented from the open-ended post workshop question from the survey (with the other likert scale items). The paper presents its purpose as demonstrating how DT can support admissions challenges and collaborative brainstorming, but then doesn't say anything about how participants experienced the process of DT through the workshop. Revisiting those 15 open-ended responses (if they got that many to write in something), would be really revealing as to how the process of participating in the workshop went. That qualitative data can add nuance to the likert data. If no one responded to the open-ended question, the authors could also state that.

Additional information regarding the responses to the open-ended question on the post-evaluation survey was added.

Finally, there are a few small editorial fixes here:

17. Line 93: Add a comma after "work"

Addressed.

18. Line 132: Add a colon after "3"

Addressed.

19. Line 186 Add an s to the word "candidate"

Addressed.

Attachment Submitted filename: Response to Reviewers.docx

# Peer review: Decision Letter 1

**DOI:** 10.1371/journal.pone.0352192.r003

**Authors:** Kendra Oliver, Andrea Cioffi

22 Apr 2026

PONE-D-25-54674R1

Leveraging design thinking to tackle contemporary admissions challenges in health professions education

PLOS One

Dear Dr. McLaughlin,

Thank you for submitting your manuscript to PLOS ONE. After careful consideration, we feel that it has merit but does not fully meet PLOS ONE’s publication criteria as it currently stands. Therefore, we invite you to submit a revised version of the manuscript that addresses the points raised during the review process.

Please see 'Additional Editor Comments' below.

Please submit your revised manuscript by Jun 06 2026 11:59PM. If you will need more time than this to complete your revisions, please reply to this message or contact the journal office at plosone@plos.org. When you're ready to submit your revision, log on to https://www.editorialmanager.com/pone/ and select the 'Submissions Needing Revision' folder to locate your manuscript file.

Please include the following items when submitting your revised manuscript:

- A letter that responds to each point raised by the academic editor and reviewer(s). You should upload this letter as a separate file labeled 'Response to Reviewers'.
- A marked-up copy of your manuscript that highlights changes made to the original version. You should upload this as a separate file labeled 'Revised Manuscript with Track Changes'.
- An unmarked version of your revised paper without tracked changes. You should upload this as a separate file labeled 'Manuscript'.

If you would like to make changes to your financial disclosure, please include your updated statement in your cover letter. Guidelines for resubmitting your figure files are available below the reviewer comments at the end of this letter.

If applicable, we recommend that you deposit your laboratory protocols in protocols.io to enhance the reproducibility of your results. Protocols.io assigns your protocol its own identifier (DOI) so that it can be cited independently in the future. For instructions see: https://journals.plos.org/plosone/s/submission-guidelines#loc-laboratory-protocols. Additionally, PLOS ONE offers an option for publishing peer-reviewed Lab Protocol articles, which describe protocols hosted on protocols.io. Read more information on sharing protocols at https://plos.org/protocols?utm_medium=editorial-email&utm_source=authorletters&utm_campaign=protocols.

As the corresponding author, your ORCID iD is verified in the submission system and will appear in the published article. PLOS supports the use of ORCID, and we encourage all coauthors to register for an ORCID iD and use it as well. Please encourage your coauthors to verify their ORCID iD within the submission system before final acceptance, as unverified ORCID iDs will not appear in the published article. *Only*  the individual author can complete the verification step; PLOS staff *cannot*  verify ORCID iDs on behalf of authors.

We look forward to receiving your revised manuscript.

Kind regards,

Andrea Cioffi

Academic Editor

PLOS One

Journal Requirements:

If the reviewer comments include a recommendation to cite specific previously published works, please review and evaluate these publications to determine whether they are relevant and should be cited. There is no requirement to cite these works unless the editor has indicated otherwise.

Please review your reference list to ensure that it is complete and correct. If you have cited papers that have been retracted, please include the rationale for doing so in the manuscript text, or remove these references and replace them with relevant current references. Any changes to the reference list should be mentioned in the rebuttal letter that accompanies your revised manuscript. If you need to cite a retracted article, indicate the article’s retracted status in the References list and also include a citation and full reference for the retraction notice.

Additional Editor Comments:

The manuscript is clearly written and well organized. The description of the workshop design, including the articulation of launching points, the structured ideation process, and the integration of design constraints (cost, geography, equity), provides a useful and potentially transferable framework for other institutions. In addition, the transparency of the methods and the availability of the dataset in a public repository represent important strengths.

However, despite these positive aspects, there are several fundamental concerns that limit the extent to which the current manuscript meets the standards required for publication.

The primary concern relates to the nature of the evidence generated. The study is based on a single, short-duration workshop involving a small, purposively selected group of participants from one institution (n=15) . The outcomes reported are largely descriptive and exploratory, consisting of generated ideas, thematic categorizations, and participants’ perceptions of the workshop experience. While these findings are informative, they do not provide empirical evidence regarding the effectiveness, feasibility, or impact of the proposed strategies.

In particular, the quantitative component is limited to self-reported perceptions measured via Likert-scale responses, which are inherently subject to response and social desirability biases. The qualitative component, although appropriate in principle, is based on heterogeneous data sources (brainstorming outputs, facilitator notes, and reflection materials), and the analytical approach, while improved, remains relatively limited in methodological depth.

A further important limitation is the absence of any evaluation of implemented interventions. Although the manuscript discusses potential strategies and prototypes generated through the DT process, it does not assess whether these strategies were adopted, nor whether they resulted in measurable improvements in admissions processes or outcomes. As such, the manuscript primarily demonstrates that design thinking can facilitate idea generation, rather than providing evidence of its effectiveness as an intervention.

More broadly, the manuscript would benefit from clearer conceptual positioning. Clarifying this positioning is essential to ensure alignment between the study design, the claims made, and the expectations of the journal.

Finally, while the conclusions are generally reasonable, they should be more explicitly grounded in the exploratory nature of the data. Statements regarding the value and utility of design thinking should be appropriately tempered to reflect the absence of outcome-based evidence.

In summary, your manuscript addresses an important topic and presents a well-described and potentially useful approach to addressing complex admissions challenges. However, substantial revisions are required to strengthen the conceptual framing, appropriately calibrate the interpretation of findings, and clarify the contribution of the study.

I would be pleased to consider a revised version of your manuscript that addresses these concerns. In particular, I encourage you to explicitly acknowledge and integrate the methodological limitations into the interpretation, and moderate claims regarding effectiveness in the absence of outcome data.

[Note: HTML markup is below. Please do not edit.]

Reviewers' comments:

Reviewer's Responses to Questions

**Comments to the Author**

1. If the authors have adequately addressed your comments raised in a previous round of review and you feel that this manuscript is now acceptable for publication, you may indicate that here to bypass the “Comments to the Author” section, enter your conflict of interest statement in the “Confidential to Editor” section, and submit your "Accept" recommendation.

Reviewer #1: All comments have been addressed

Reviewer #2: All comments have been addressed

**********

2. Is the manuscript technically sound, and do the data support the conclusions?

The manuscript must describe a technically sound piece of scientific research with data that supports the conclusions. Experiments must have been conducted rigorously, with appropriate controls, replication, and sample sizes. The conclusions must be drawn appropriately based on the data presented.

Reviewer #1: (No Response)

Reviewer #2: Yes

**********

3. Has the statistical analysis been performed appropriately and rigorously?

Reviewer #1: Yes

Reviewer #2: Yes

**********

4. Have the authors made all data underlying the findings in their manuscript fully available?

The PLOS Data policy requires authors to make all data underlying the findings described in their manuscript fully available without restriction, with rare exception (please refer to the Data Availability Statement in the manuscript PDF file). The data should be provided as part of the manuscript or its supporting information, or deposited to a public repository. For example, in addition to summary statistics, the data points behind means, medians and variance measures should be available. If there are restrictions on publicly sharing data—e.g. participant privacy or use of data from a third party—those must be specified.

Reviewer #1: Yes

Reviewer #2: Yes

**********

5. Is the manuscript presented in an intelligible fashion and written in standard English?

PLOS ONE does not copyedit accepted manuscripts, so the language in submitted articles must be clear, correct, and unambiguous. Any typographical or grammatical errors should be corrected at revision, so please note any specific errors here.

Reviewer #1: Yes

Reviewer #2: Yes

**********

6. Review Comments to the Author

Please use the space provided to explain your answers to the questions above. You may also include additional comments for the author, including concerns about dual publication, research ethics, or publication ethics. (Please upload your review as an attachment if it exceeds 20,000 characters)

Reviewer #1: Thank you for addressing my comments! I wish you good luck to your admissions leadership and team with the adjustments to your process.

Reviewer #2: Thank you for addressing the original points! You do have a repeat sentence on Line 190 to remove. Thank you for this work!

**********

7. PLOS authors have the option to publish the peer review history of their article (what does this mean?). If published, this will include your full peer review and any attached files.

If you choose “no”, your identity will remain anonymous but your review may still be made public.

**Do you want your identity to be public for this peer review?**  For information about this choice, including consent withdrawal, please see our Privacy Policy.

Reviewer #1: **Yes:** Emil T. Chuck, Ph.D., Health Professional Student Association

Reviewer #2: No

**********

[NOTE: If reviewer comments were submitted as an attachment file, they will be attached to this email and accessible via the submission site. Please log into your account, locate the manuscript record, and check for the action link "View Attachments". If this link does not appear, there are no attachment files.]

To ensure your figures meet our technical requirements, please review our figure guidelines: https://journals.plos.org/plosone/s/figures

You may also use PLOS’s free figure tool, NAAS, to help you prepare publication quality figures: https://journals.plos.org/plosone/s/figures#loc-tools-for-figure-preparation.

NAAS will assess whether your figures meet our technical requirements by comparing each figure against our figure specifications.

# Author comment: Author response to Decision Letter 2

**DOI:** 10.1371/journal.pone.0352192.r004

29 May 2026

Response to Academic Editor comments

1. The primary concern relates to the nature of the evidence generated. The study is based on a single, short-duration workshop involving a small, purposively selected group of participants from one institution (n=15).

We acknowledge this limitation and have addressed it throughout the manuscript by clarifying the exploratory nature of the study, the purposive sampling approach, and the limited generalizability associated with a single, small sample, short-duration workshop conducted at one institution.

2. The outcomes reported are largely descriptive and exploratory, consisting of generated ideas, thematic categorizations, and participants’ perceptions of the workshop experience. While these findings are informative, they do not provide empirical evidence regarding the effectiveness, feasibility, or impact of the proposed strategies.

We have clarified throughout the manuscript that the purpose of this work was to inform early-stage strategy development and gather stakeholder insights, rather than to assess intervention outcomes or establish evidence of impact.

3. In particular, the quantitative component is limited to self-reported perceptions measured via Likert-scale responses, which are inherently subject to response and social desirability biases. The qualitative component, although appropriate in principle, is based on heterogeneous data sources (brainstorming outputs, facilitator notes, and reflection materials), and the analytical approach, while improved, remains relatively limited in methodological depth.

We have further acknowledged these methodological limitations throughout the manuscript, including the reliance on self-reported perceptions and the use of multiple qualitative sources, and clarified the scope and interpretive boundaries of the analysis presented.

4. A further important limitation is the absence of any evaluation of implemented interventions. Although the manuscript discusses potential strategies and prototypes generated through the DT process, it does not assess whether these strategies were adopted, nor whether they resulted in measurable improvements in admissions processes or outcomes. As such, the manuscript primarily demonstrates that design thinking can facilitate idea generation, rather than providing evidence of its effectiveness as an intervention.

We have acknowledged throughout the manuscript that the study did not include implementation or outcome evaluation of the proposed strategies and clarified that the work was intended to demonstrate the utility of the design thinking process for stakeholder engagement and idea generation.

5. More broadly, the manuscript would benefit from clearer conceptual positioning. Clarifying this positioning is essential to ensure alignment between the study design, the claims made, and the expectations of the journal.

We have clarified the conceptual positioning of the study throughout the manuscript to better align the study aims, design, and conclusions with the exploratory scope of the work.

6. Finally, while the conclusions are generally reasonable, they should be more explicitly grounded in the exploratory nature of the data. Statements regarding the value and utility of design thinking should be appropriately tempered to reflect the absence of outcome-based evidence.

We have revised the conclusions throughout the manuscript to more clearly reflect the exploratory nature of the findings and to ensure that the statements regarding the value and utility of design thinking are appropriately contextualized within the scope of the evidence presented.

7. In summary, your manuscript addresses an important topic and presents a well-described and potentially useful approach to addressing complex admissions challenges. However, substantial revisions are required to strengthen the conceptual framing, appropriately calibrate the interpretation of findings, and clarify the contribution of the study.

We have made substantial revisions throughout the manuscript to strengthen the conceptual framing, better align the interpretation of the findings with the exploratory nature of the study, and clarify the overall contribution of the work.

Attachment Submitted filename: Response to Academic Editor comments.docx

# Editor report: Decision Letter 2

**DOI:** 10.1371/journal.pone.0352192.r005

**Authors:** Kendra Oliver, Andrea Cioffi, Andrea Cioffi

7 Jun 2026

Leveraging design thinking to tackle contemporary admissions challenges in health professions education

PONE-D-25-54674R2

Dear Dr. McLaughlin,

We’re pleased to inform you that your manuscript has been judged scientifically suitable for publication and will be formally accepted for publication once it meets all outstanding technical requirements.

Within one week, you’ll receive an e-mail detailing the required amendments. When these have been addressed, you’ll receive a formal acceptance letter and your manuscript will be scheduled for publication.

An invoice will be generated when your article is formally accepted. Please note, if your institution has a publishing partnership with PLOS and your article meets the relevant criteria, all or part of your publication costs will be covered. Please make sure your user information is up-to-date by logging into Editorial Manager at Editorial Manager® and clicking the ‘Update My Information' link at the top of the page. For questions related to billing, please contact billing support.

If your institution or institutions have a press office, please notify them about your upcoming paper to help maximize its impact. If they’ll be preparing press materials, please inform our press team as soon as possible -- no later than 48 hours after receiving the formal acceptance. Your manuscript will remain under strict press embargo until 2 pm Eastern Time on the date of publication. For more information, please contact onepress@plos.org.

Kind regards,

Andrea Cioffi

Academic Editor

PLOS One

Additional Editor Comments (optional):

Reviewers' comments:

# Editor report: Acceptance letter

**DOI:** 10.1371/journal.pone.0352192.r006

**Authors:** Kendra Oliver, Andrea Cioffi, Andrea Cioffi

PONE-D-25-54674R2

PLOS One

Dear Dr. McLaughlin,

I'm pleased to inform you that your manuscript has been deemed suitable for publication in PLOS One. Congratulations! Your manuscript is now being handed over to our production team.

At this stage, our production department will prepare your paper for publication. This includes ensuring the following:

* All references, tables, and figures are properly cited

* All relevant supporting information is included in the manuscript submission,

* There are no issues that prevent the paper from being properly typeset

You will receive further instructions from the production team, including instructions on how to review your proof when it is ready. Please keep in mind that we are working through a large volume of accepted articles, so please give us a few days to review your paper and let you know the next and final steps.

Lastly, if your institution or institutions have a press office, please let them know about your upcoming paper now to help maximize its impact. If they'll be preparing press materials, please inform our press team within the next 48 hours. Your manuscript will remain under strict press embargo until 2 pm Eastern Time on the date of publication. For more information, please contact onepress@plos.org.

You will receive an invoice from PLOS for your publication fee after your manuscript has reached the completed accept phase. If you receive an email requesting payment before acceptance or for any other service, this may be a phishing scheme. Learn how to identify phishing emails and protect your accounts at https://explore.plos.org/phishing.

If we can help with anything else, please email us at customercare@plos.org.

Thank you for submitting your work to PLOS ONE and supporting open access.

Kind regards,

PLOS ONE Editorial Office Staff

on behalf of

Dr. Andrea Cioffi

Academic Editor

PLOS One
